# A study on the influence of athletes' psychological safety on training persistence and its chain mediation mechanism

**DOI:** 10.3389/fpsyg.2026.1787593

**Published:** 2026-04-22

**Authors:** Xuedan Xu

**Affiliations:** Department of Physical Education, Wuxi Taihu University, Wuxi, China

**Keywords:** chain mediation, cognitive fatigue, psychological safety, training enjoyment, training persistence

## Abstract

**Introduction:**

As competitive training shifts from a traditional “load-optimization” paradigm toward an integrated “load-experience-development” framework, explaining training persistence requires greater attention to athletes' situational interaction and subjective experience. Drawing on psychological safety theory, the self-regulatory resource model, and self-determination theory, this study proposed a chained pathway model linking psychological safety, cognitive fatigue, training enjoyment, and training persistence.

**Methods:**

Questionnaire data were collected from active athletes (*N* = 526). Structural equation modeling (SEM) was used to test the hypothesized relationships among psychological safety, cognitive fatigue, training enjoyment, and training persistence. Multi-group comparisons were further conducted to examine whether the focal pathways differed across endurance, skill, and strength/competition disciplines.

**Results:**

The results showed that psychological safety was negatively associated with cognitive fatigue and positively associated with training enjoyment. Cognitive fatigue and training enjoyment were, in turn, significantly associated with training persistence, and the sequential pathway from psychological safety to training persistence through reduced cognitive fatigue and enhanced training enjoyment was supported. In addition, the magnitude of the focal pathways varied across discipline types, indicating that the relationship between training interaction conditions and sustained engagement was context-dependent.

**Discussion:**

These findings suggest that training persistence should not be understood merely as a manifestation of individual willpower. Rather, it reflects the combined influence of cognitive resource expenditure and affective reward, both of which are shaped by enduring interaction patterns within the training environment. The study extends current explanations of training persistence and offers practical implications for fostering psychologically safe training climates, calibrating load-recovery rhythms, and strengthening process-oriented motivation in high-performance sport settings.

## Introduction

In recent years, the governance orientation of competitive sports training systems has gradually shifted from a traditional “load-centered” paradigm toward a more integrated framework emphasizing athlete welfare and performance synergy. Within this evolving context, athletes' subjective experiences, psychological states, and social interaction environments have increasingly been recognized as critical determinants of training participation quality and long-term persistence (Kidman, 2005; Bourdon et al., 2017).

Historically, competitive training has been dominated by a physiological load paradigm, in which training management primarily focuses on optimizing training intensity, volume, and recovery cycles to maximize physiological adaptation and athletic performance. Within this framework, coaching practices have largely concentrated on monitoring physical workload and recovery status, while comparatively less attention has been devoted to athletes' psychological experiences and relational dynamics within training environments. However, as issues such as athlete burnout, overtraining, and early sport withdrawal have attracted growing scholarly attention, recent research has increasingly emphasized the importance of training experiences, psychological states, and social interactions in sustaining long-term training engagement (Meeusen et al., 2013; Gustafsson et al., 2017).

Training persistence represents not only a necessary condition for transforming training programs into competitive performance but also a critical psychological and behavioral foundation for preventing burnout, dropout, and non-functional overtraining (Meeusen et al., 2013; Gustafsson et al., 2017). Emerging evidence suggests that in prolonged high-pressure and high-load training environments, athletes' persistence in training is shaped not solely by external incentives or performance outcomes, but also by their perceptions of respect, understanding, and psychological safety within the training context (Walton et al., 2024). In this sense, the training environment should not be viewed merely as a space for physical conditioning and technical development; rather, it also constitutes a social arena in which relational structures and organizational culture are manifested (Edmondson, 1999; Jowett et al., 2023). Consequently, a key challenge for contemporary competitive training systems lies in maintaining the physiological demands of high-performance sport while simultaneously cultivating psychologically supportive environments that promote sustained athlete engagement (Rice et al., 2022).

In training contexts characterized by high workloads and dense performance evaluations, athletes are frequently required to perform technical attempts and pursue skill advancement under conditions of visible comparison and the risk of failure. Research indicates that when training environments are dominated by humiliating feedback, unilateral command structures, and zero-tolerance expectations, athletes often adopt strategies of silence and superficial compliance in order to maintain interpersonal harmony, while concealing genuine difficulties and concerns during daily interactions (Bartholomew et al., 2009). Under such circumstances, the absence of psychological safety does not necessarily manifest as overt conflict. Instead, it gradually becomes internalized as reluctance to express uncertainty, reduced willingness to experiment with new techniques, and emotional disengagement from interpersonal and institutional relationships. As a result, training persistence can no longer be adequately explained solely through goal-setting or external motivation. Rather, it increasingly depends on whether athletes believe that within the training environment they can safely express vulnerability and be genuinely understood and accepted (Rice et al., 2022; Jowett et al., 2023).

It should also be noted that training interaction structures may vary across different institutional sport systems. In some highly market-oriented professional sport systems, athletes typically possess greater autonomy in career decisions and training arrangements, and coach–athlete relationships tend to be more decentralized. In contrast, China's competitive sports system is characterized by a relatively centralized and institutionally structured training framework in which athletes undergo long-term systematic development within organized training environments. Within this context, the interpersonal climate of training teams and the relational dynamics between coaches and athletes may exert a more direct influence on athletes' psychological experiences. Consequently, examining the mechanisms through which psychological safety operates in training environments is particularly important for understanding athletes' participation and persistence behaviors in such institutionalized settings.

Training enjoyment also plays a pivotal role in the development of persistent training behavior (Kendzierski and DeCarlo, 1991; Williams, 2008). Positive emotional experiences enhance the immediate perceived value of training activities and provide intrinsic rewards that help buffer the pressures associated with high workload and performance risk. In turn, these experiences encourage athletes to repeatedly choose continued participation in training over extended periods. Within training environments, psychological safety, cognitive fatigue, and training enjoyment coexist and interact dynamically. A lack of psychological safety may intensify athletes' long-term cognitive resource consumption, leading to the accumulation of cognitive fatigue. Such fatigue can undermine enjoyment during training, and the resulting shift toward a negative emotional tone may ultimately translate into disengagement and reduced persistence in training behavior (Marcora et al., 2009; Van Cutsem et al., 2017; Taylor et al., 2022). Taken together, training persistence should be understood as the outcome of an integrated process involving organizational climate, situational perception, cognitive load, and emotional experience.

Existing research on training persistence has predominantly focused on classical motivational constructs such as achievement motivation, self-efficacy, and goal orientation (Bandura, 1997; Bartholomew et al., 2009). Comparatively limited attention has been devoted to examining how power structures and interaction climates within training environments influence athletes' sustained participation through mediating mechanisms such as psychological safety. Although recent studies have begun to address variables such as cognitive fatigue and training enjoyment, these investigations often rely on short-term performance fluctuations or transient psychological states. Consequently, they have not yet established a unified framework capable of explaining the sequential mechanism linking situational safety perception, cognitive resource depletion, emotional experience, and behavioral persistence (Marcora et al., 2009; Van Cutsem et al., 2017; Williams, 2008). This gap is particularly evident in highly controlled and authority-oriented training contexts, where the absence of psychological safety is often normalized under narratives of strict discipline and mental toughness, leaving its potential effects on cognitive load and emotional foundations insufficiently examined (Taylor et al., 2022; Walton et al., 2024).

To address these theoretical and practical gaps, the present study focuses on athletes within competitive training environments. Psychological safety is conceptualized as a core indicator of athletes' perceptions of the training climate. Cognitive fatigue and training enjoyment are introduced as key mediating mechanisms linking situational experiences with behavioral outcomes. Based on this framework, the study constructs and tests a chained mediation model involving psychological safety, cognitive fatigue, training enjoyment, and training persistence. Using questionnaire data and structural equation modeling, the study seeks to clarify how psychological safety within training environments influences athletes' training persistence through the sequential transformation of cognitive resource consumption and emotional experience. In doing so, the research aims to extend existing motivation-based explanations by providing a more comprehensive perspective grounded in situational, cognitive, and emotional mechanisms.

## Theoretical foundations and research hypotheses

### Theoretical foundations

#### Theoretical foundations of psychological safety: the psychological safety theory in organizational behavior

Psychological safety emerged from organizational behavior research and was formally articulated by Edmondson in the late 1990s. It denotes a shared belief that individuals can voice concerns, ask questions, and acknowledge mistakes in group settings without incurring humiliation, punishment, or negative interpersonal evaluation (Edmondson, 1999; Newman et al., 2017). At its core, the construct captures a relational climate in which interpersonal risk-taking is perceived as low-cost, allowing individuals to disclose uncertainty and vulnerability without fear of identity devaluation or social exclusion. Subsequent scholarship has further refined psychological safety as a trust-embedded relational judgment centered on whether “speaking truth is safe” and whether “attempting failure is permitted” (Frazier et al., 2017).

Competitive training environments are characterized by public evaluation, repetitive trial-and-error, and ongoing social comparison—conditions under which athletes routinely confront skill deficits and intermittent setbacks (Saxe and Hardin, 2022). When such environments institutionalize unilateral control, zero tolerance for error, and humiliating feedback, athletes are more likely to minimize interpersonal exposure through silence, compliance, and emotional suppression, thereby constraining authentic communication and discouraging technical risk-taking. By contrast, training climates that normalize mistakes, encourage inquiry, and provide constructive feedback foster athletes' willingness to disclose uncertainty, seek support proactively, and persist in mastering complex skills. In this regard, everyday micro-behaviors—such as requesting demonstrations after failed attempts, reporting physical discomfort or psychological strain, voicing concerns about training plans, or endorsing the belief that failure does not imply incompetence—function as behavioral indicators of psychological safety appraisals in routine practice. These moment-to-moment choices help determine whether athletes adopt merely performative compliance or sustain genuine engagement across high-intensity training cycles (Jowett et al., 2023; Saxe and Hardin, 2022).

Taken together, psychological safety offers a context-sensitive framework for interpreting variability in training behavior through the lens of organizational climate and relational dynamics. It illuminates the latent psychological costs embedded in authoritarian training cultures and provides a conceptual foundation for explaining the situational origins of athletes' training persistence. Treating psychological safety as a core perceptual index of the training environment also enables a more systematic account of how safety climates shape long-term participation by altering cognitive resource expenditure and affective experience, thereby grounding the chained mediation model advanced in this study.

#### Explanation of cognitive exhaustion mechanisms: the self-regulation resource model

The self-regulation resource model was advanced by Baumeister et al. (1998) in the late 1990s. The model posits that self-control relies on a finite pool of regulatory resources that are consumed when individuals modulate affect, inhibit impulses, and override inertia. When these regulatory operations are repeatedly engaged over a short period, subsequent self-control capacity declines, rendering individuals less persistent and more vulnerable to distraction and interference (Baumeister and Vohs, 2007). Resource depletion is further accelerated when individuals face concurrent goal conflict and affective strain, because maintaining goal-consistent behavior requires sustained engagement of higher-order regulatory functions to suppress competing impulses. Within this framework, cognitive fatigue represents a salient subjective correlate of depletion, typically characterized by sustained mental effort, difficulty maintaining attention, and reduced cognitive flexibility. Behaviorally, it is expressed as attentional lapses, attenuated task involvement, and heightened sensitivity to disengagement cues (Hagger et al., 2010).

Competitive training is rarely a single-task demand; rather, it constitutes a complex performance ecology marked by continuous evaluation, stringent time constraints, and intersecting role expectations. Athletes must repeatedly inhibit withdrawal impulses, tolerate discomfort, comply with coaching directives, and regulate affective responses to errors and setbacks, all of which require sustained mobilization of self-control resources (Van Cutsem et al., 2017). When psychological safety is low, athletes must also devote cognitive capacity to monitoring others' evaluations and calibrating self-presentation to avoid negative interpersonal consequences, thereby increasing both the intensity and frequency of regulatory expenditure. In applied settings, depletion commonly appears as increased boredom, greater distractibility by task-irrelevant stimuli, and diminished sensitivity to technical cues. Athletes may complete prescribed workloads while exhibiting low-quality engagement, reflected in reduced concentration, less proactive problem-solving, and constrained technical experimentation. Over time, they may default to conservative execution strategies to minimize error exposure, and sustained functioning in this depleted mode can consolidate into chronic disengagement and structural erosion of training persistence (Englert, 2017; Marcora et al., 2009).

In sum, the self-regulation resource model offers a mechanistic account of cognitive fatigue in training contexts. By conceptualizing cognitive fatigue as a functional decrement following prolonged, high-intensity deployment of regulatory resources, it clarifies how high-control, high-pressure environments progressively undermine athletes' regulatory capacity and, in turn, compromise their ability to sustain high-quality engagement during demanding training cycles.

#### Linking emotions to perseverance behavior: self-determination theory

Self-determination theory (SDT) was introduced by Deci and Ryan in the 1980s and subsequently consolidated into a comprehensive motivational framework around 2000 to account for qualitative differences in motivation and their implications for behavioral persistence (Deci and Ryan, 2000). SDT posits three universal basic psychological needs—autonomy, competence, and relatedness. Autonomy reflects the experience of volition and self-endorsement in one's actions; competence denotes perceived effectiveness and capability in pursuing valued activities; relatedness refers to feeling accepted, cared for, and connected within significant relationships or groups. When these needs are adequately satisfied, individuals are more likely to internalize goals and develop autonomous forms of motivation, accompanied by heightened vitality, enjoyment, and perceived meaning. Conversely, chronic need thwarting undermines autonomous motivation, amplifies negative affect, and increases vulnerability to burnout, withdrawal, and merely compliant participation (Ryan and Deci, 2017; Vallerand, 1997).

In competitive training contexts, SDT provides a rigorous account of how affective experience translates into sustained behavioral engagement. Training enjoyment should not be treated as a fleeting emotional state; rather, it functions as an affective signature of autonomous motivation. When athletes appraise training as self-endorsed, experience opportunities to demonstrate competence, and perceive supportive relational conditions, their affective tone becomes more positive and the activity's intrinsic value is strengthened. This motivational configuration promotes sustained engagement even under demanding load cycles. By contrast, training climates dominated by external control, punitive regulation, and comparative pressure frustrate autonomy and relatedness, thereby attenuating enjoyment and reframing training as externally imposed. Under such conditions, athletes become more susceptible to emotional exhaustion and behavioral disengagement (Ntoumanis, 2001; Pelletier et al., 1995).

In sum, SDT specifies a coherent sequence from basic need satisfaction to autonomous motivation and enhanced affective experience, culminating in more persistent behavior. Integrating SDT into the present chained mediation model clarifies how psychologically safe training climates may elevate enjoyment by supporting autonomy and relatedness, thereby strengthening stable training persistence.

### Research hypotheses

Building on the foregoing theoretical foundations, this study investigates athletes' psychological safety in training contexts and specifies cognitive fatigue and training enjoyment as central mechanisms linking situational appraisals to behavioral outcomes. Accordingly, we propose a sequential pathway model—Psychological Safety → Cognitive Fatigue → Training Enjoyment → Training Persistence—and derive the following hypotheses.

#### H1: direct effects of psychological safety on training-related states

Psychological safety is expected to reduce anticipated interpersonal risk and defensive self-monitoring during training, thereby preserving cognitive resources and creating contextual conditions conducive to positive affective experience. In line with the preceding analysis, its functional implications are primarily reflected in reduced cognitive strain and enhanced training enjoyment.

**H1a:** Psychological safety is negatively associated with athletes' cognitive fatigue.
**H1b:** Psychological safety is positively associated with athletes' training enjoyment.

#### H2: effects of cognitive fatigue on affective experience and persistence

The self-regulation resource model suggests that sustained, high-intensity self-regulation generates resource depletion, which is subjectively experienced as cognitive fatigue. Such fatigue undermines the hedonic value of ongoing activity and compromises the capacity to sustain engagement. In high-load training cycles, accumulated cognitive fatigue is therefore expected to diminish training enjoyment and weaken the propensity to persist under stress.

**H2a:** Cognitive fatigue is negatively associated with training enjoyment.
**H2b:** Cognitive fatigue is negatively associated with training persistence.

#### H3: effects of training enjoyment on training persistence

Self-determination theory posits that autonomous motivation—often expressed through enjoyment and vitality—provides an affective–motivational foundation for sustained behavior. Higher training enjoyment should increase the perceived intrinsic value of training, encouraging repeated voluntary investment and more stable persistence over time. Within this framework, training enjoyment is expected to function both as a direct predictor of persistence and as a key affective mechanism through which psychological safety exerts its influence.

**H3a:** Training enjoyment is positively associated with training persistence.
**H3b:** Training enjoyment mediates the relationship between psychological safety and training persistence.

#### H4: indirect effect of psychological safety on training persistence via cognitive fatigue

In high-load environments characterized by dense evaluation, insufficient psychological safety is likely to intensify self-presentational monitoring and interpersonal risk management. The combined demands of task execution and expressive control increase regulatory expenditure, thereby elevating cognitive fatigue. As cognitive fatigue accumulates, athletes' attentional quality, willingness to attempt, and long-term commitment may deteriorate, establishing a cognitive pathway linking psychological safety to training persistence.

**H4a:** Cognitive fatigue mediates the relationship between psychological safety and training persistence.

#### H5: serial mediation and discipline-based heterogeneity

Integrating the above propositions, psychological safety is expected to reduce cognitive fatigue by curbing unnecessary regulatory expenditure. Lower cognitive fatigue should, in turn, restore the intrinsic appeal and positive affective tone of training, thereby enhancing training enjoyment and ultimately strengthening training persistence. This yields a serial mechanism: Psychological Safety → Cognitive Fatigue → Training Enjoyment → Training Persistence. Moreover, because sport disciplines differ in load structure, technical complexity, and training rhythm, the magnitude of this serial pathway may vary across discipline types.

**H5a:** Psychological safety exerts an indirect positive effect on training persistence through reduced cognitive fatigue and increased training enjoyment.
**H5b:** Sport discipline type moderates the strength of the serial mediating pathways.

**Figure 1:**
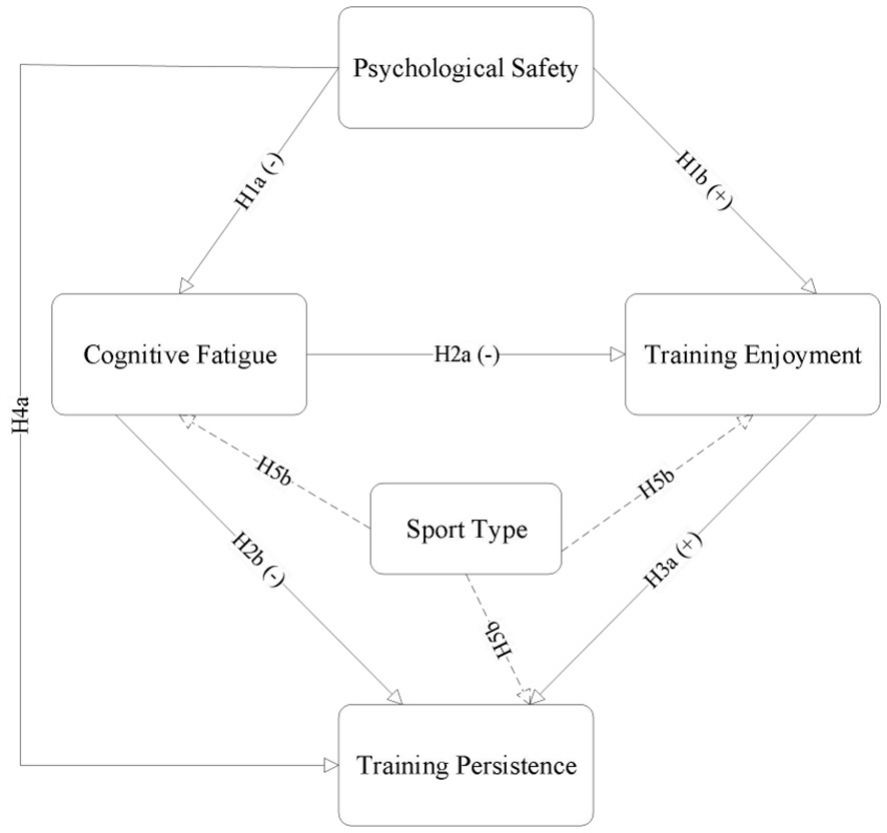
Conceptual model of the study.

Taken together, the hypothesized model is summarized in Figure 1.

## Research design

### Study population and sample sources

This study employed a multistage stratified cluster sampling strategy to recruit athletes currently embedded in China's competitive sport training system. Participants were drawn primarily from university elite sport teams and provincial/municipal professional teams, covering multiple sport categories and training stages. This sampling frame was selected to test, within a unified institutional context, the mechanisms linking psychological safety, cognitive fatigue, training enjoyment, and training persistence. Given their sustained exposure to high-intensity training and dense evaluative scrutiny, this population provides an appropriate context for observing how situational factors crystallize into training-related behavioral patterns.

Geographically, the sample covered North China, East China, Central China, and Southwest China and included nine provincial-level administrative regions: Beijing, Tianjin, Shandong, Jiangsu, Hubei, Hunan, Sichuan, Yunnan, and Guangdong. Restricting recruitment to a single national context minimized cross-cultural confounding and enabled a more focused examination of the relationships among psychological safety, cognitive fatigue, training enjoyment, and training persistence within the same training system and organizational culture. This relative contextual homogeneity strengthens internal validity and improves the interpretability of model estimates.

Individual athletes served as the unit of analysis. A total of 620 questionnaires were distributed, and 589 were returned. Of these, 63 were excluded due to invariant responding, excessive missing data, or aberrant completion times, yielding 526 valid cases and an effective response rate of 89.3%. Prior to the main survey, a pilot test with 50 athletes was conducted to refine ambiguous wording and standardize administration instructions and response procedures, thereby improving measurement clarity and overall data quality.

Prior to the formal investigation, this study was approved by the Ethics Review Committee of Wuxi Taihu University (Approval Number: HREC-2025-074). This study involved a questionnaire survey of human participants, with all individuals voluntarily participating after providing informed consent. The research strictly adhered to the Declaration of Helsinki and relevant ethical guidelines, ensuring anonymity throughout the survey process. Data were used solely for academic research purposes, and results were presented without any identifiable personal information.

The final sample comprised athletes aged 16–25 years (*M* = 19.8, *SD* = 2.1), including 289 males (55.0%) and 237 females (45.0%). By sport category, 184 athletes (35.0%) participated in endurance sports, 176 (33.5%) in skill-based sports, and 166 (31.5%) in strength/contact sports. Regarding technical classification, 121 athletes (23.0%) held National Level 1 certification, 228 (43.4%) held National Level 2 certification, and 177 (33.6%) reported no certification. Mean training history was 7.3 years (*SD* = 2.9), and weekly training frequency was concentrated between five and eight sessions.

### Measurement instruments

This study used a structured questionnaire to assess four focal constructs: psychological safety, cognitive fatigue, training enjoyment, and training persistence. All items were rated on a five-point Likert scale, and composite scores were computed as the mean of the corresponding items. Higher scores indicate higher levels of the target construct.

#### Psychological safety

Psychological safety was used to assess athletes' subjective sense of security when expressing genuine feelings, acknowledging mistakes, or voicing dissent during training. As existing scales did not adequately address the specific contextual needs of this study, no validated sport-specific instruments were employed. To accurately capture athletes' psychological experiences during high-intensity training, the study developed five items based on Edmondson's (1999) psychological safety framework, tailored to the competitive training context. These items encompass key dimensions such as “safety of expression,” “acceptance of mistakes,” and “fear of humiliation or punishment.” For example: “During training, I can honestly express my difficulties without fear of negative evaluation” and “When I make mistakes in training, teammates and coaches respond in a helpful rather than humiliating manner.” Items were rated on a 1 = strongly disagree to 5 = strongly agree scale, with the mean serving as the psychological safety score. Confirmatory factor analysis revealed significant standardized factor loadings exceeding 0.60 (*p* < 0.001) for all items, indicating strong internal consistency and convergent validity (Cronbach's α = 0.83, CR = 0.85, AVE = 0.53).

#### Cognitive fatigue

Cognitive fatigue assessed athletes' subjective experience of cognitive exhaustion during high-intensity training and sustained self-regulatory demands, with emphasis on attentional maintenance difficulties, increased mental effort, slowed processing, and reduced perceived control. Drawing on prior work linking psychological fatigue to self-regulatory resources (Hagger et al., 2010; Marcora et al., 2009) and supplemented by athlete interviews to capture context-relevant expressions, the study developed a six-item measure. Representative items included: “I need to exert significant mental effort to maintain focus during training” and “I often feel slowed reactions and unclear thinking during training.” Items were rated from 1 (strongly disagree) to 5 (strongly agree), and the mean item score was used as the cognitive fatigue index. CFA results showed that all standardized loadings were significant and exceeded 0.60 (*p* < 0.001). Internal consistency and convergent validity were acceptable (Cronbach's α = 0.85, CR = 0.87, AVE = 0.52), indicating that the measure reliably captures athletes' cognitive exhaustion in training settings.

#### Training enjoyment

Training enjoyment reflected athletes' positive affect and intrinsic reward during training and was conceptualized as an affective indicator of motivation quality. The measure drew on the core structure of the Physical Activity Enjoyment Scale (PACES) (Kendzierski and DeCarlo, 1991), with item wording streamlined and adapted for competitive training, resulting in eight items. Sample items included: “Overall, I feel enjoyment during training” and “Even when training intensity is high, I still derive positive experiences from it.” Responses ranged from 1 (strongly disagree) to 5 (strongly agree), and the mean of the items served as the training enjoyment score. CFA supported the scale's measurement quality, with all standardized loadings significant and above 0.60 (*p* < 0.001). Reliability and convergent validity were strong (Cronbach's α = 0.88, CR = 0.89, AVE = 0.57), supporting its capacity to capture athletes' positive affective tone during training.

#### Training persistence

Training persistence captured athletes' propensity to sustain adherence to training plans over time, particularly their behavioral commitment to remain engaged despite fatigue, stress, or limited short-term reinforcement. Scale development began with a systematic review of established measures of sport commitment, persistence, and adherence (Scanlan et al., 1993; Wilson et al., 2004). From this review, recurrent behavioral indicators were synthesized, including completing training as scheduled, minimizing absences or early termination, maintaining commitment during setbacks or performance plateaus, and proactively sustaining training quality. These indicators were then translated into an initial item pool tailored to the competitive training context. Content validity was evaluated by three domain experts in sport training and sport psychology, who rated and refined items in terms of importance, clarity, and contextual relevance; items with strong consensus were retained. A small-sample pilot test subsequently identified and removed redundant or weak items using item-level diagnostics and preliminary structural analyses. The final instrument comprised six items assessing timely completion of training, perseverance under challenge, and sustained engagement quality. Sample items included: “Even when feeling tired or under significant pressure, I strive to complete training as planned” and “When encountering setbacks in training, I do not readily reduce my training commitment.” Responses were recorded on a five-point Likert scale (1 = strongly disagree to 5 = strongly agree), and the mean of the six items served as the training persistence score. Confirmatory factor analysis supported the measure's unidimensionality, with all standardized loadings significant and exceeding 0.60 (*p* < 0.001). Reliability and convergent validity were acceptable (Cronbach's α = 0.84, CR = 0.86, AVE = 0.52), indicating that the scale reliably captures athletes' training persistence.

#### Sport category and covariates

Athletes self-reported their primary sport, which was categorized into endurance, skill-based, and strength/competitive disciplines based on load structure and technical characteristics. This classification served as the grouping variable for examining structural heterogeneity in the serial mechanism (psychological safety → cognitive fatigue → training enjoyment → training persistence) across sport contexts. Age, gender, years of training, and weekly training frequency were included as covariates in subsequent models to reduce potential confounding attributable to demographic differences and training exposure.

#### Measurement quality assurance

Prior to the full survey, a pilot study evaluated semantic clarity, completion time, and score distributions. Experts in sport psychology and training science reviewed and refined the item content. Across all measures, internal consistency and convergent validity met recommended thresholds, with Cronbach's α values above 0.80, CR values exceeding 0.82, and AVE values consistently above 0.52, providing a sound measurement basis for the subsequent structural equation modeling analyses.

Additionally, athletes self-reported their primary sport category, which was further classified based on differences in energy metabolism characteristics and movement patterns across sports. Referencing a common classification framework in sports training science (Bompa and Haff, 2009), the sample sports were categorized into endurance sports, skill-based sports, and strength/competitive sports to examine structural differences in the psychological safety–cognitive fatigue–training enjoyment–training persistence pathway under varying training contexts. Age, gender, years of training experience, and weekly training frequency were incorporated as control variables in subsequent model analyses to mitigate confounding effects of demographic characteristics and training exposure on core pathways. Prior to the full-scale survey, a pilot study with a small sample examined item semantic clarity, response time, and scoring distribution. Experts in sports psychology and training science reviewed and revised the scale content. Overall results showed that all scales achieved Cronbach's α above 0.80, CR exceeding 0.82, and AVE above 0.52, providing a robust measurement foundation for subsequent structural equation modeling.

To ensure measurement accuracy and cultural adaptability, all scales used in this study underwent translation/back-translation procedures. Translation was performed by two native Chinese speakers, with consistency confirmed through back-translation. Content validity was assessed by inviting three sports psychology experts to evaluate item relevance, clarity, and applicability. The average expert rating was 4.5 out of 5, indicating high content validity.

### Data analysis methods

Data management and statistical analyses were conducted in SPSS 26.0 and Mplus 8.3. Prior to inferential testing, the raw dataset was screened for missingness and outliers, and responses exhibiting invariance or logical inconsistency were removed. Distributional characteristics were examined to evaluate normality assumptions. Descriptive statistics (means and standard deviations) and Pearson correlations were then computed for all focal variables to summarize bivariate associations and inform subsequent structural equation modeling (SEM).

Potential common method variance (CMV) was evaluated using multiple complementary procedures. First, Harman's single-factor test was applied to all measurement items. A single-factor model was estimated and compared with the theorized multi-factor structure using global fit indices. Second, a latent method factor was specified in Mplus to further probe CMV. CMV was considered unlikely to bias substantive conclusions when the single-factor model did not account for an unusually large proportion of covariance and when the method-factor specification did not yield a meaningful improvement in model fit.

Construct validity was examined via confirmatory factor analysis (CFA). A four-factor measurement model—psychological safety, cognitive fatigue, training enjoyment, and training persistence—was specified as the baseline structure, and a single-factor model as well as alternative competing models were estimated for comparison. Model fit was evaluated using χ^2^/*df* , the Comparative Fit Index (CFI), the Tucker–Lewis Index (TLI), the Root Mean Square Error of Approximation (RMSEA), and the Standardized Root Mean Square Residual (SRMR). Convergent validity was assessed through standardized factor loadings, composite reliability (CR), and average variance extracted (AVE). Discriminant validity was evaluated using the Fornell–Larcker criterion by comparing the square root of each construct's AVE with its correlations with other latent variables. Structural analyses proceeded only after the measurement model demonstrated acceptable fit and adequate convergent and discriminant validity.

Hypothesized structural relations were tested using SEM. Consistent with the theoretical framework, a serial mediation model was specified with psychological safety as the exogenous predictor, cognitive fatigue and training enjoyment as mediators, and training persistence as the endogenous outcome. Age, gender, years of training, and weekly training frequency were included as covariates to reduce confounding attributable to demographic differences and training exposure. Parameters were estimated using maximum likelihood with robust standard errors (MLR) to accommodate potential minor departures from multivariate normality. Standardized path coefficients and corresponding significance tests were reported. Indirect and serial indirect effects were evaluated using bias-corrected bootstrapping with 5,000 resamples and 95% confidence intervals; mediation was inferred when the confidence interval excluded zero (Hayes, 2018). To examine whether the serial mechanism differed by sport discipline, multi-group SEMs were estimated for endurance, skill-based, and strength/competitive groups. Group differences were tested by progressively constraining focal paths to equality across groups and comparing constrained and unconstrained models using χ^2^ difference tests. All hypothesis tests were two-tailed with α = 0.05, and results are reported as model fit indices, standardized estimates, and confidence intervals.

## Research findings

### Descriptive statistics and correlation analysis

To characterize the distributional properties of the study variables and establish preliminary association patterns, descriptive statistics, and Pearson correlation analyses were conducted for the focal constructs. Table 1 reports the means and standard deviations for all primary variables. All indicators were standardized, and the resulting distributions satisfied conventional normality criteria, with no evidence of extreme skewness or influential outliers. These diagnostics indicate that the dataset is suitable for subsequent structural modeling.

**Table 1:** Descriptive statistics of study variables (*N* = 526).

Variable	Mean	Standard deviation
Psychological safety	3.60	0.50
Cognitive fatigue	2.80	0.58
Training enjoyment	3.70	0.48
Training persistence	3.50	0.49

As shown in Table 1, athletes reported moderately high psychological safety during training (*M* = 3.60, *SD* = 0.50), suggesting that the training climate generally permits the expression of concerns and difficulties. Cognitive fatigue was comparatively low (*M* = 2.80, *SD* = 0.58), indicating relatively limited perceived cognitive strain in the sample. Training enjoyment was high (*M* = 3.70, *SD* = 0.48), reflecting a predominantly positive affective tone during training. Training persistence was also relatively high (*M* = 3.50, *SD* = 0.49), suggesting sustained commitment and continuity in training engagement despite challenges.

**Figure 2:**
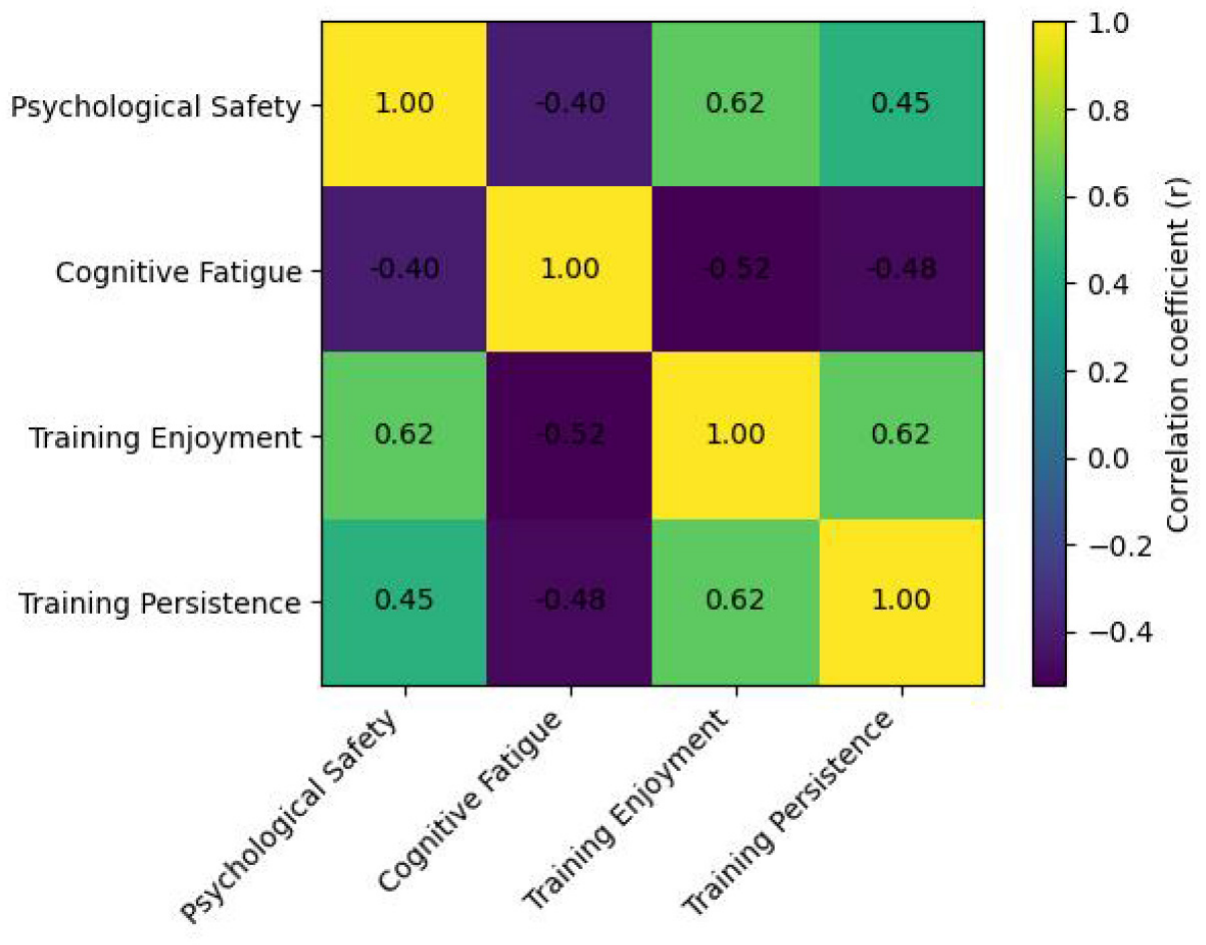
Pearson correlation matrix of the latent variables.

Correlation analyses (see Figure 2) indicated that psychological safety was positively associated with training enjoyment (*r* = 0.62, *p* < 0.001) and training persistence (*r* = 0.45, *p* < 0.001). These patterns imply that athletes in psychologically safer environments tend to report greater enjoyment and demonstrate stronger persistence, consistent with psychological safety theory emphasizing the role of interpersonal risk reduction in sustaining engagement (Edmondson, 1999). Psychological safety was also negatively associated with cognitive fatigue (*r* = −0.40, *p* < 0.001), indicating that safer climates are linked to lower perceived cognitive depletion.

Cognitive fatigue, in turn, exhibited negative associations with training enjoyment (*r* = −0.52, *p* < 0.001) and training persistence (*r* = −0.48, *p* < 0.001), suggesting that elevated cognitive fatigue is accompanied by diminished positive affect and reduced behavioral continuity. Training enjoyment was positively related to training persistence (*r* = 0.62, *p* < 0.001), further underscoring the affective basis of sustained participation.

Taken together, the descriptive and correlational results show a coherent pattern in which psychological safety covaries positively with training enjoyment and training persistence, whereas cognitive fatigue covaries negatively with both outcomes. These preliminary associations provide empirical grounding for subsequent tests of the hypothesized mediation mechanisms.

### Measurement model validation: confirmatory factor analysis (CFA)

Prior to conducting confirmatory factor analysis (CFA), the potential influence of common method bias was first examined. Harman's single-factor test indicated that the first unrotated factor accounted for 32.6% of the total variance, which is below the commonly accepted threshold of 40%. This result suggests that common method bias was unlikely to pose a substantial threat to the validity of the data.

To further assess this issue, a single-factor model was estimated using CFA. The results revealed poor model fit (χ^2^/*df* = 8.71, CFI = 0.63, TLI = 0.59, RMSEA = 0.112, SRMR = 0.094), which was markedly inferior to that of the proposed four-factor measurement model. Subsequently, a latent method factor was incorporated into the model to further evaluate potential method bias. The inclusion of this factor did not lead to a meaningful improvement in model fit, indicating that common method variance exerted only a negligible influence on the observed relationships. Taken together, these findings suggest that common method bias does not represent a serious concern in the present study.

Building on this assessment, confirmatory factor analysis was conducted to evaluate the reliability and validity of the measurement scales. As shown in Table 2, Cronbach's alpha coefficients, composite reliability (CR), and average variance extracted (AVE) were calculated for all core latent constructs. The results indicate strong internal consistency across all constructs, with Cronbach's alpha values exceeding the recommended threshold of 0.80. In addition, composite reliability values for psychological safety (0.90), cognitive fatigue (0.89), training enjoyment (0.93), and training persistence (0.91) were all above 0.80, further supporting the reliability of the measurement instruments.

**Table 2:** Confirmatory factor analysis results.

Latent variable	Cronbach α	CR	AVE
Psychological safety	0.90	0.92	0.35
Cognitive fatigue	0.89	0.96	0.49
Training enjoyment	0.93	0.94	0.36
Training persistence	0.91	0.93	0.36

Although the AVE values for the latent constructs ranged from 0.35 to 0.49 and therefore fell below the conventional benchmark of 0.50, this outcome may be partially attributable to sample characteristics and measurement context. Importantly, the CFA results showed that all standardized factor loadings were statistically significant and exceeded 0.60 (*p* < 0.001). Moreover, the overall model fit indices satisfied recommended criteria (χ^2^/*df* = 2.24, CFI = 0.96, TLI = 0.95). Accordingly, the convergent validity of the constructs was considered acceptable despite the relatively low AVE values. In addition, the heterotrait–monotrait (HTMT) ratios among latent constructs were all below 0.85, providing further evidence of satisfactory discriminant validity. For indicators exhibiting relatively lower factor loadings, additional analyses were conducted to confirm their theoretical relevance to the corresponding constructs, thereby ensuring the robustness of the measurement model.

The overall fit of the measurement model was subsequently evaluated, and the results are reported in Table 3. All fit indices met established evaluation standards, indicating an adequate model fit. Specifically, the χ^2^/*df* ratio was 2.24, which falls below the recommended upper limit of 3.00. The comparative fit index (CFI = 0.96) and Tucker–Lewis index (TLI = 0.95) both exceeded the commonly accepted threshold of 0.90, indicating strong comparative fit. The root mean square error of approximation (RMSEA = 0.05) was well below the recommended cutoff of 0.06, suggesting low model misfit, while the standardized root mean square residual (SRMR = 0.04) was also below the recommended threshold of 0.08, further supporting the adequacy of the measurement model.

**Table 3:** Model fit indices for measurement model.

Fit index	Model value	Recommended threshold
*χ^2^*/*df*	2.24	< 3.00
CFI	0.96	>0.90
TLI	0.95	>0.90
RMSEA	0.05	< 0.06
SRMR	0.04	< 0.08

In summary, the CFA results demonstrate that the measurement model exhibits satisfactory reliability and validity. The findings confirm the appropriateness of the latent construct structure and provide a solid methodological foundation for the subsequent structural model analysis.

### Structural path analysis

After ensuring the measurement model possessed good reliability and fit, this study constructed a structural path model to examine the structural relationships among latent variables. The structural model specified a serial pathway—psychological safety → cognitive fatigue → training enjoyment → training persistence—and included relevant background variables as covariates to reduce potential confounding.

**Figure 3:**
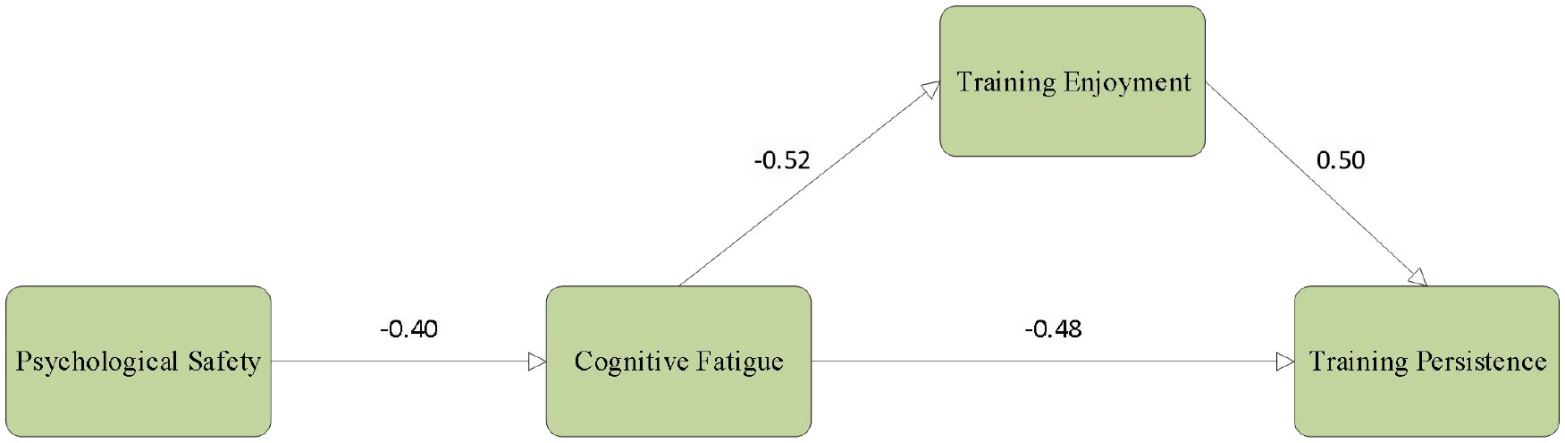
Standardized path coefficients in the SEM.

As shown in Figure 3, psychological safety was negatively associated with cognitive fatigue (β = −0.40, *p* < 0.001), indicating that higher perceived safety corresponds to lower cognitive depletion during training. Cognitive fatigue, in turn, was negatively related to training enjoyment (β = −0.52, *p* < 0.001) and training persistence (β = −0.48, *p* < 0.001), suggesting that elevated cognitive fatigue undermines positive affect and weakens sustained engagement. These findings are consistent with the self-regulation resource perspective, which links heightened regulatory demands to impaired affective experience and reduced behavioral persistence (Baumeister et al., 1998). Training enjoyment was positively associated with training persistence (β = 0.50, *p* < 0.001), indicating that positive affective experience contributes to more stable commitment across prolonged training cycles.

Bootstrap mediation analyses further supported the hypothesized indirect pathway. Psychological safety exerted a significant indirect effect on training persistence via cognitive fatigue and training enjoyment [β_indirect = 0.18, 95% CI (0.11, 0.27)], with the confidence interval excluding zero. Collectively, these results support the proposed serial mediation mechanism and underscore psychological safety as an upstream contextual resource that promotes training persistence by attenuating cognitive fatigue and enhancing training enjoyment.

### Moderating effects analysis

The comparative analysis revealed that different sport disciplines exert distinct influences on the pathway linking psychological safety, cognitive fatigue, training enjoyment, and training persistence. Table 4 presents the corresponding path coefficients for each group.

**Table 4:** Multi-group SEM results.

Project type	Path	Standardized coefficient (β)	Δ*χ^2^* (*df* = 1)	Significance
Endurance	Psychological Safety → Cognitive Fatigue	−0.40	4.21	*p* < 0.01
	Cognitive Fatigue → Training Enjoyment	−0.45	6.29	*p* < 0.001
	Cognitive Fatigue → Training Persistence	−0.48	5.12	*p* < 0.01
	Training Enjoyment → Training Persistence	0.40	4.13	*p* < 0.02
Skill	Psychological Safety → Cognitive Fatigue	−0.35	4.02	*p* < 0.03
	Cognitive Fatigue → Training Enjoyment	−0.40	5.98	*p* < 0.02
	Cognitive Fatigue → Training Persistence	−0.45	4.89	*p* < 0.02
	Training Enjoyment → Training Persistence	0.45	3.79	*p* < 0.04
Strength_Contact	Psychological Safety → Cognitive Fatigue	−0.25	3.65	*p* < 0.05
	Cognitive Fatigue → Training Enjoyment	−0.50	4.48	*p* < 0.001
	Cognitive Fatigue → Training Persistence	−0.38	4.01	*p* < 0.03
	Training Enjoyment → Training Persistence	0.38	2.91	*p* < 0.03

The effect of psychological safety on cognitive fatigue differed across sport disciplines. Specifically, the standardized path coefficient was −0.40 (*p* < 0.001) for endurance sports, −0.35 (*p* < 0.02) for skill-based sports, and −0.25 (*p* < 0.05) for strength and combat sports. These findings indicate that psychological safety consistently reduces cognitive fatigue across all disciplines, although the magnitude of this effect varies. Among the three categories, the relationship is strongest in endurance sports.

Cognitive fatigue exhibited a significant negative association with both training enjoyment and training persistence across all sport types. In endurance sports, cognitive fatigue was negatively related to training enjoyment (β = −0.45, *p* < 0.001) and training persistence (β = −0.48, *p* < 0.02). In skill-based sports, the corresponding effects were β = −0.40 (*p* < 0.02) for training enjoyment and β = −0.45 (*p* < 0.04) for training persistence. In strength and combat sports, cognitive fatigue reduced training enjoyment (β = −0.50, *p* < 0.001) and training persistence (β = −0.38, *p* < 0.03). Overall, these results demonstrate that cognitive fatigue plays a consistently detrimental role in shaping both affective responses and persistence behaviors in training contexts, although the magnitude of these effects varies across disciplines. Notably, the influence on training enjoyment is most pronounced in strength and combat sports.

Training enjoyment, in turn, showed a significant positive effect on training persistence in all sport categories. The standardized path coefficient was 0.40 (*p* < 0.02) for endurance sports, 0.45 (*p* < 0.04) for skill-based sports, and 0.38 (*p* < 0.03) for strength and combat sports. These findings highlight training enjoyment as a crucial determinant of sustained training engagement. The relationship appears particularly strong in skill-based sports, where enjoyment exerts the most substantial motivational influence on persistence.

Taken together, the results indicate that the structural relationships among psychological safety, cognitive fatigue, training enjoyment, and training persistence remain directionally consistent across sport disciplines. However, the strength of these relationships varies across groups, reflecting discipline-specific differences in athletes' psychological responses and behavioral engagement within training environments. These findings provide empirical insight into the contextualized psychological mechanisms underlying training persistence across diverse sport settings.

## Discussion

### Mediating effect of psychological safety on training persistence

The structural model results reveal a significant negative relationship between psychological safety and cognitive fatigue, indicating that higher levels of psychological safety are associated with lower levels of cognitive fatigue (β = −0.40, *p* < 0.001). In addition, psychological safety demonstrates significant positive associations with both training enjoyment and training persistence. These findings provide empirical support for the proposed sequential pathway linking psychological safety, cognitive fatigue, training enjoyment, and training persistence. Specifically, a psychologically secure training environment appears to simultaneously alleviate athletes' cognitive resource depletion and enhance their positive emotional experiences, thereby fostering sustained behavioral engagement in training over time.

Nevertheless, several methodological considerations should be acknowledged when interpreting these results. The sequential pathway examined in this study was estimated using structural equation modeling based on cross-sectional questionnaire data. While structural equation modeling allows for the examination of theoretically specified relationships among latent variables, cross-sectional research designs inherently capture statistical associations rather than temporal or causal processes. Consequently, the pathway “psychological safety → cognitive fatigue → training enjoyment → training persistence” should be interpreted as a theoretically grounded relational structure supported by empirical evidence, rather than as definitive proof of causal mechanisms. Future research should therefore employ longitudinal designs or experimental interventions to further examine the temporal ordering and causal dynamics underlying these psychological processes. Such approaches would enable a more rigorous evaluation of how psychological safety within training environments shapes athletes' cognitive experiences, emotional responses, and sustained training engagement over time.

This pattern can be interpreted at the convergence of psychological safety theory and self-regulation perspectives. Psychological safety reflects individuals' appraisals of the interpersonal consequences of taking social risks (Edmondson, 1999; Frazier et al., 2017). In highly controlled and evaluative training environments, athletes who perceive low psychological safety may engage in continuous impression management, heightened monitoring of others' reactions, and suppression of authentic feelings. Such sustained “interpersonal vigilance” imposes substantial regulatory costs. From the standpoint of the self-regulation resource model, repeated emotional suppression and self-presentational control consume limited regulatory resources, which is experienced subjectively as attentional diffusion and cognitive fatigue (Baumeister et al., 1998; Hagger et al., 2010). The observed negative association between psychological safety and cognitive fatigue provides empirical support for this resource-depletion account. When athletes believe that mistakes, questions, and expressions of discomfort will not elicit punitive responses, defensive self-monitoring diminishes and cognitive resources can be redirected from error avoidance toward task-focused execution and learning, thereby facilitating persistence through more efficient allocation of psychological resources.

Training enjoyment constitutes the affective conduit linking a safe climate to long-term commitment within the proposed chain. Self-determination theory posits that satisfaction of autonomy and relatedness needs provides a foundation for positive affect and higher-quality motivation (Deci and Ryan, 2000; Ryan and Deci, 2017). Psychological safety supports these needs by legitimizing voice and participation, enabling athletes to communicate candidly about physical and psychological states, and fostering experiences of understanding and acceptance within coach–athlete and teammate relationships. In turn, high-intensity training becomes less an externally imposed requirement and more a self-endorsed investment, which strengthens enjoyment and, ultimately, stabilizes training persistence.

### How cognitive fatigue diminishes training enjoyment and behavioral continuity

The model results indicate that cognitive fatigue is negatively associated with training enjoyment, such that higher cognitive fatigue corresponds to lower enjoyment. Cognitive fatigue also exerts a significant inhibitory effect on training persistence, with a path coefficient of moderate magnitude. When training is repeatedly appraised as sustained “mental effort” and “cognitive overdraft,” enjoyment tends to deteriorate first; over time, this affective depletion is followed by an erosion of stable behavioral commitment. In this sense, enjoyment and persistence appear to be jointly shaped by a common process of regulatory resource consumption and motivational recalibration.

From the perspective of the self-control resource model, cognitive fatigue reflects the cumulative consequence of prolonged, high-load deployment of self-regulatory resources. Competitive training requires athletes to sustain attention across consecutive sessions and cycles, inhibit impulses to disengage, regulate affective reactions to errors and setbacks, and continue implementing technical–tactical intentions despite pain and somatic fatigue (Baumeister et al., 1998; Hagger et al., 2010). The aggregation of these demands keeps regulatory resources chronically engaged. When such engagement persists in the absence of adequate recovery, training ceases to be construed as a manageable challenge and is increasingly experienced as an additional burden. Subjectively, this shift is expressed as a transition from focused involvement to psychological weariness. Consequently, even when training tasks are inherently meaningful or potentially rewarding, their intrinsic appeal is readily offset by the perception that the required psychological expenditure is no longer sustainable. Athletes may therefore redefine training in terms of endurance—“getting through it” or “surviving this phase”—and enjoyment becomes difficult to maintain.

Self-determination theory further clarifies why enjoyment is particularly vulnerable under conditions of elevated cognitive fatigue. SDT posits that high-quality motivation and positive affect depend on the satisfaction of autonomy, competence, and relatedness needs (Deci and Ryan, 2000; Ryan and Deci, 2017). When cognitive fatigue is high, athletes often experience a persistent mismatch between environmental demands and their perceived capacity to meet them, which progressively undermines competence. Training-related decisions then become more externally regulated and less volitional, weakening autonomy. Under resource strain, sensitivity to external feedback is heightened, whereas the felt sense of agency and control is attenuated; autonomy and competence needs are therefore more likely to remain unmet. Training accordingly shifts from an activity that is self-endorsed and experienced as attainable to an obligation that must be completed. Enjoyment no longer stems from skill development and self-realization but is overshadowed by depletion, frustration, and affective blunting. The observed negative association between cognitive fatigue and training enjoyment can thus be interpreted as a quantitative indicator of need frustration in the training context.

At the behavioral level, integrating the self-control resource model with SDT elucidates the pathways through which cognitive fatigue undermines training persistence. Persistence is sustained through repeated decisions that implicitly trade off psychological costs against expected benefits. As cognitive fatigue accumulates, continued training entails additional regulatory demands layered onto an already taxed system, raising the perceived marginal cost of “one more set” or an extended cycle. In contrast, anticipated benefits—such as performance improvement, selection opportunities, and self-realization—are typically delayed and uncertain. Under such a cost–benefit structure, reducing investment, engaging in minimal compliance, or even discontinuing participation may be experienced as a rational strategy to conserve self-regulatory resources. The significant negative path from cognitive fatigue to training persistence identified in this study reflects this progressive shift in choice, whereby training gradually moves from an activity perceived as worth sustained investment toward an experience construed as a situation one seeks to terminate as quickly as possible.

### The critical role of training enjoyment in training persistence

The findings show that training enjoyment exerts a significant positive effect on training persistence, indicating that persistence is not solely propelled by external constraints or delayed outcome rewards. Rather, it is anchored in athletes' affective appraisal of the training process. Enjoyment marks training as an activity worthy of repeated investment, thereby shifting sustained participation from passive compliance to a more stable self-regulatory mode.

From a self-determination theory perspective, enjoyment can be conceptualized as an affective signature of basic psychological need satisfaction. When training environments support autonomy, competence, and relatedness, positive affect provides immediate feedback regarding motivational quality and signals deeper internalization of training goals as self-endorsed pursuits (Deci and Ryan, 2000; Ryan and Deci, 2017). Athletes thus persist in repetitive, monotonous, and high-risk practice because the process continuously generates experiences of progress, control, and interpersonal support. Over time, these experiences consolidate into enjoyment and become a reproducible psychological return that sustains ongoing participation.

Enjoyment contributes to persistence partly by recalibrating athletes' cost–benefit evaluations. Benefits in competitive training are typically delayed and uncertain, whereas costs are immediate and salient, including fatigue, pain, failure exposure, and evaluative risk. Enjoyment functions as an immediate yet non-contingent reward, enabling athletes to maintain perceived value under high-cost input while reducing the salience of disengagement options. In this way, enjoyment reframes training from a purely depleting obligation to an investment that yields proximal affective returns, thereby increasing the likelihood of repeated engagement across training cycles.

Training enjoyment may also operate through a motivational threshold mechanism. When positive affect is insufficient, training is more likely to become instrumentalized and enacted in a formalistic manner. Behavioral participation may be maintained, but engagement quality deteriorates, technical risk-taking and proactive exploration diminish, and the long-term accumulation of competence and self-efficacy is weakened. This dynamic can generate a self-reinforcing cycle of low-quality participation, low returns, and progressively lower enjoyment. Conversely, when enjoyment is consistently activated, athletes are more likely to sustain proactive engagement and an exploratory orientation. This enhances immediate practice quality and, through sustained competence experiences, stabilizes future willingness to invest. Persistence thus becomes a durable psychological–behavioral configuration rather than a short-lived reliance on willpower.

### Moderating role of sport type on the pathway between psychological safety and training behavior

Multi-group SEM results demonstrate that the serial chain linking psychological safety, cognitive fatigue, training enjoyment, and training persistence is directionally invariant across sport disciplines, yet its effect magnitudes vary systematically. This pattern indicates that psychological safety does not function as a uniform contextual resource across training environments. Instead, its influence is embedded in discipline-specific load structures, technical demands, and risk configurations, yielding pronounced contextual dependence. Psychological safety, therefore, should not be treated as an abstract, universally transferable asset; rather, it is instantiated and amplified through the distinctive psychological cost structures that characterize different sports.

In endurance disciplines, the buffering effect of psychological safety on cognitive fatigue is particularly salient. Endurance training typically involves prolonged duration, high repetitiveness, and limited immediate feedback, requiring sustained self-regulation under monotonous load and slow performance gains. Under these conditions, psychological strain often stems less from discrete performance episodes than from chronic uncertainty regarding whether continued endurance is worthwhile. Psychological safety appears to reduce self-doubt and relational tension, thereby enabling athletes to allocate cognitive resources more efficiently to pacing control and the management of physiological fatigue. Consequently, when psychological safety is compromised, its cumulative deficits are more likely to manifest as pronounced cognitive fatigue in endurance contexts.

In skill-based disciplines, training enjoyment emerges as a stronger proximal driver of training persistence. These sports require fine-grained motor control, iterative error correction, and deliberate technical risk-taking, and training commonly entails frequent failure and public comparison. The capacity to derive positive affect from technical experimentation and incremental progress therefore becomes central to sustained engagement. Psychological safety attenuates the implicit equation of failure with incompetence, preserving relational and emotional space for athletes to attempt novel skills, disclose limitations, and engage in repeated refinement. Training enjoyment subsequently internalizes this safety as a positive affective orientation toward practice, such that persistence is sustained by an emotional climate in which exploration and error are legitimate.

In strength and combat disciplines, the detrimental effect of cognitive fatigue on both enjoyment and persistence is especially pronounced. These contexts often involve elevated physical risk, intense competition, and immediate outcome feedback. Athletes must tolerate substantial physical load while simultaneously regulating affect and inhibiting impulses within confrontational interpersonal dynamics. When psychological safety is low, defensive vigilance and self-control demands escalate, and cognitive resources may be rapidly exhausted. Once cognitive fatigue accumulates, training becomes less intrinsically attractive and enjoyment deteriorates more quickly than in other disciplines, thereby exerting a more direct erosive influence on persistence.

Overall, the findings suggest that the psychological costs “saved” by psychological safety are discipline-contingent: in some contexts safety primarily reduces long-term self-regulatory burden, in others it protects the affective space required for technical exploration, and in still others it buffers cognitive overdraft under high-risk confrontation. These multi-group results do not diminish the general relevance of psychological safety; rather, they specify the contextualized nature of its pathways and underscore that explanations of training persistence must situate psychological mechanisms within the logic of particular sport disciplines.

## Conclusions and recommendations

### Conclusions

Drawing on psychological safety theory, the self-control resource model, and self-determination theory, this study developed and validated a serial process model linking situational psychological safety to training persistence through cognitive fatigue and training enjoyment. The model elucidates the generative logic of persistence in competitive training and specifies how this mechanism is contextually differentiated across sport disciplines. The principal conclusions are summarized as follows.

(1) Psychological safety serves as a foundational contextual determinant of athletes' training states. Higher psychological safety was consistently associated with lower cognitive strain and more positive training experiences, suggesting that safety is not merely an auxiliary affective condition but a subjective crystallization of training interaction structures and evaluative practices. When the training climate legitimizes voice, tolerates errors, and minimizes humiliating risk, athletes are more likely to allocate attention to task execution rather than defensive self-monitoring, thereby establishing more sustainable psychological conditions for continued engagement.
(2) Cognitive fatigue constitutes a central cognitive channel through which training persistence deteriorates, concurrently undermining affective experience and behavioral maintenance. Cognitive fatigue not only attenuated training enjoyment but also directly weakened persistence, implying that attrition in persistence cannot be attributed solely to physical load or external pressure. Instead, it reflects functional degradation resulting from chronically mobilized self-regulatory resources. When training is repeatedly experienced as intense mental effort and difficulty sustaining attention, positive experiences become harder to generate and participation is more likely to drift toward formalistic compliance and low-quality engagement.
(3) Training enjoyment represents a pivotal affective hub in the consolidation of training persistence. Enjoyment exhibited a robust positive association with persistence, indicating that persistence is not simply the prolongation of willpower expenditure but depends on the capacity of training to generate intrinsic rewards. Enjoyment provides a reproducible source of positive reinforcement, increasing athletes' willingness to invest and sustaining engagement quality under recurrent stress and fatigue, thereby stabilizing persistence as a relatively enduring behavioral tendency.
(4) The influence of psychological safety on training persistence is transmitted through a clear serial mechanism: psychological safety indirectly promotes long-term persistence by reducing cognitive fatigue and enhancing training enjoyment. This finding underscores that situational experience does not map directly onto behavioral outcomes; rather, its effects crystallize through successive transformations in regulatory resource expenditure and affective appraisal.
(5) Sport discipline type meaningfully moderates the strength of the core pathways, revealing stable directional relations but discipline-contingent effect magnitudes. Multi-group analyses identified distinct sensitivity points across the links of “safety buffering fatigue,” “fatigue eroding enjoyment,” and “enjoyment sustaining persistence.” In endurance disciplines, time accumulation and delayed feedback amplify the importance of safety climate in shaping resource expenditure. In skill-based disciplines, dense trial-and-error and frequent ability exposure heighten the persistence value of enjoyment as an affective return. In strength and combat disciplines, elevated risk and confrontational structure intensify the detrimental impact of cognitive fatigue on both affect and persistence. These results indicate that persistence mechanisms should be interpreted and implemented within discipline-specific logics rather than extrapolated as homogeneous generalizations.

### Recommendations

Based on the above conclusions, the following recommendations are proposed:

(1) Strengthen supportive communication practices within training interactions. During training, coaches' feedback strategies and communication styles play a crucial role in shaping athletes' perceptions of the training environment. Coaches should prioritize task-oriented and improvement-focused feedback by offering explicit guidance on technical deficiencies and corresponding developmental strategies. At the same time, feedback should avoid directly associating training errors with judgments of personal ability. In addition, structured opportunities for questioning and discussion should be incorporated into routine training sessions and periodic performance reviews. Such practices enable athletes to articulate technical difficulties and training concerns more openly, thereby reducing perceived interpersonal risk within the training environment.
(2) Emphasize the balanced allocation of cognitive load in training program design. Beyond physiological demands, training requires sustained attention, emotional regulation, and continuous decision-making, all of which consume substantial cognitive resources. When organizing training cycles, coaches should avoid repeatedly imposing complex technical tasks and intense evaluative pressure during prolonged high-intensity phases. Instead, training programs should incorporate task segmentation, adaptive pacing strategies, and scheduled recovery intervals. These adjustments allow athletes to maintain training intensity while also preserving essential cognitive resources, thereby supporting sustained high-quality engagement in training activities.
(3) Enhance the affective value of training through strengthened process-oriented feedback. Over extended training periods, athletes' emotional engagement is often shaped by their perceptions of progress and goal clarity. Coaches can enhance the motivational appeal of training by establishing clearly defined, stage-specific objectives and providing timely feedback on technical improvements. Such feedback enables athletes to recognize incremental progress in skill development and performance capacity. Furthermore, when appropriate, allowing athletes a degree of participation in auxiliary training arrangements or recovery strategy selection—without compromising overall training objectives—may foster a stronger sense of agency and commitment within the training process.
(4) Implement differentiated training management strategies based on sport-specific characteristics. Different sports vary substantially in training rhythm, technical complexity, and competitive intensity. Accordingly, training management practices should account for the ways in which sport-specific contexts shape athletes' psychological experiences. In endurance sports, periodic goal feedback and adjustments to training pace may help alleviate psychological fatigue associated with prolonged repetitive practice. In skill-based disciplines, maintaining a relatively relaxed training climate can facilitate continuous technical exploration through trial and error. In strength and combat sports, greater attention should be given to emotional regulation during training interactions to prevent excessive competitive pressure from undermining the overall training experience.
(5) Strengthen the cultivation of training environment management competencies within coach education systems. Coach education and continuing professional development programs should incorporate targeted instruction on communication strategies, feedback delivery, and the management of training climates. The use of case-based learning and scenario simulations may help coaches develop more effective management strategies for high-intensity training environments. Such initiatives can provide institutional support for the creation of psychologically supportive and development-oriented training atmospheres.

## Limitations and directions for future research

Although this study identified the structural pathway through which psychological safety influences athletes' training persistence within a situational–cognitive–emotional framework, several limitations should be acknowledged.

First, the study employed a cross-sectional questionnaire design. Although structural equation modeling enabled the examination of theoretically specified relationships among variables, cross-sectional data inherently capture statistical associations rather than temporal or causal processes. Consequently, the causal direction of the proposed pathway cannot be rigorously established. Future research should therefore adopt longitudinal tracking designs or experimental intervention approaches to further examine the dynamic mechanism linking psychological safety, cognitive fatigue, training enjoyment, and training persistence.

Second, the data were primarily derived from athletes' self-reported questionnaires. Although statistical procedures were conducted to assess common method bias, the possibility of residual measurement homogeneity cannot be entirely excluded. To enhance the robustness of future findings, subsequent studies should incorporate multi-source data collection strategies, including coach evaluations, training records, and objective behavioral indicators.

Finally, the sample consisted mainly of athletes operating within China's competitive sports training system. Institutional arrangements and training cultures vary considerably across national sport systems, which may influence the structural relationships between psychological safety and training-related behaviors. Future research should therefore conduct cross-cultural comparative investigations to evaluate the broader applicability and contextual generalizability of the proposed model.

## Data Availability

The raw data supporting the conclusions of this article will be made available by the authors, without undue reservation.
